# CAESAR’s legacy: a new era of rigor in preclinical studies of cardioprotection

**DOI:** 10.1007/s00395-021-00874-8

**Published:** 2021-05-20

**Authors:** Roberto Bolli

**Affiliations:** Institute of Molecular Cardiology, University of Louisville, 550 S. Jackson Street, 3rd Floor, ACB, Louisville, KY 40202 USA

This year marks the golden jubilee of the field of cardioprotection (defined here as limitation of myocardial infarct [MI] size). Fifty years ago, in January 1971, Dr. Braunwald and colleagues published a landmark paper (cited > 1700 times) in which they showed that indices of myocardial injury could be improved (or worsened) by changing the conditions of the heart *after* the onset of coronary artery occlusion [23]. It was a revolutionary idea, for at that time, it was thought that the extent of cell death in the tissue distal to an occluded coronary artery was fixed and unchangeable. The 1971 paper [23] was the genesis of a new field that would spawn tens of thousands of publications over the next 50 years. Many readers of this editorial were not even born at that time, but I distinctly remember the excitement that the article sparked in the cardiovascular community. Intuitively, it was exhilarating to think that myocardium destined to die could still be salvaged after the onset of MI. The excitement was substantiated by the observation that, in survivors of MI, the amount of dead myocardium was a major determinant of subsequent morbidity and mortality [26]. Therefore, reducing infarct size should improve clinical outcome after MI.

The seminal work of Braunwald et al. [23] motivated many an investigator, including myself, to identify cardioprotective therapies in experimental models of MI. It was a massive effort. Over the past half century, thousands of drugs or interventions have been claimed to reduce infarct size in various animal models [4, 13, 14]. Unfortunately, with the notable exception of early reperfusion, none of these allegedly cardioprotective therapies has made it into the clinical realm, and today we still do not have a drug that is approved for limitation of infarct size in patients with acute MI. When one considers the gargantuan efforts and resources invested in attempting to limit infarct size, the outcome has been very disappointing indeed. Certainly, it was not for lack of trying. So, what went wrong?

Probably the first meeting specifically devoted to looking into this issue, and particularly into lack of reproducibility and translational failure in cardioprotection, was envisioned and spearheaded by the author of this editorial and convened in Bethesda, MD, under the auspices of the National Heart, Lung, and Blood Institute (NHLBI), in 2003 [4]. The Working Group concluded that the failure to develop infarct-sparing treatments with proven clinical efficacy had multifarious causes. Insufficient rigor was the leading culprit, as discussed below, but there were also other factors. Animal models do not faithfully recapitulate the clinical setting of acute MI. This is quite obvious when one considers the differences between adolescent/young animals with no comorbidities and middle-aged or elderly patients with multiple comorbidities and on multiple medications. The number of variables in the clinical setting is vastly greater than that in the experimental setting. Many patients have intermittent coronary occlusions prior to acute MI, undergo gradual or intermittent reperfusion, or have subtotal coronary occlusion, all of which modify infarct size [15]. The protective effects of many therapies are likely small, and thus difficult to detect with clinically available methods, such as magnetic resonance imaging or plasma troponin levels.

Furthermore, the remarkable improvement in the prognosis of acute MI in the last 3 decades [1, 31] (resulting from early reperfusion and better medical therapy) has made it increasingly difficult to show clinical benefit not only with infarct-sparing interventions, but with any new therapy. The incidence of ST- segment elevation MI (STEMI) (the type of MI where infarct size reduction is most likely to make a difference) has decreased dramatically, by > 70% [7, 31]. Thus, fewer and fewer patients are candidates for infarct-sparing treatments. And among patients with STEMI, clinical outcome has improved dramatically. Many recent trials have found that, in the first year after STEMI, mortality was < 2% and the rate of hospital admission for heart failure averaged only 3% (range, 0–7%) [1]. A striking example is the recent BAMI trial in patients with STEMI (mostly [86%] anterior) and left ventricular dysfunction (average ejection fraction = 39%) [24]. In this high-risk population, at 2 years, mortality was 3.8% (instead of the expected 12%) and the incidence of MACE (cardiovascular death or hospitalization for heart failure) was 9.7%. Clearly, the number of patients necessary to show a statistically significant improvement in these numbers would be prohibitive. And in most cases, the expected benefit is not striking: a reduction in infarct size of, say. 25% of the risk region would salvage < 10% of the left ventricle, which probably would not be enough to improve clinical outcome. In summary, the need for cardioprotection is decreasing. As cardiovascular medicine advances, the number of acute MI patients in whom infarct-sparing therapies can be shown to make a difference continues to shrink.

This, however, does not mean that cardioprotection is no longer necessary—only that it is increasingly hard to demonstrate its utility, and that the target population must be carefully selected. There is still, and there will always be, a subset of patients who develop severe heart failure, particularly those with a large anterior MI or multiple, recurrent MIs; in this minority, the ability to limit infarct size would likely improve prognosis. This, I believe, is the reason why research on cardioprotection has continued for 50 years despite countless translational failures. That public and private entities continue to spend large amounts of money in clinical trials aimed at limiting infarct size is an eloquent testimony to a persisting, but unmet, need.

Nevertheless, doing the same thing over and over again and expecting a different result would meet Einstein’s famous definition of insanity. As pointed out in the 2003 Bethesda meeting [4] and subsequently echoed by several other groups and investigators [6, 9, 11–13, 19, 22, 25, 27, 29], if efforts to achieve cardioprotection are to be fruitful, a fundamental change of approach is needed. The 2003 Working Group concluded that the most important cause for the failure of cardioprotective therapies to be translated was the lack of rigor in preclinical studies, which has led to spurious and irreproducible results at the experimental level and premature and “negative” clinical trials [4]. Pursuant to the recommendations of the Working Group, the CAESAR (**C**onsortium for preclinic**A**l ass**ES**sment of c**AR**dioprotective therapies) infrastructure was established in 2010 [17, 21]. The mission of this multicenter, NHLBI-supported consortium was to form a public infrastructure, available to all investigators, that would test putative cardioprotective therapies with the same standards of rigor used in multicenter clinical trials. In particular, the studies conducted by the CAESAR investigators adhered to the following criteria: protocols were carefully standardized and strictly adhered to; animals were randomized; investigators were blinded; data analysis was conducted by independent, blinded cores (a pathology core for infarct size measurement and a biomarker core for troponin measurements); criteria for inclusion and exclusion of animals were established a priori (i.e., before the study) and not changed during or after the study; rigorous statistical analysis was performed by an independent statistician; the number of animals to be studied was established a priori (i.e., before beginning the studies) based on power calculations; therapies were tested in three different species (mice, rabbits, and pigs); and the same protocol was conducted in two different laboratories to verify reproducibility and assess the effects of local factors (e.g., technician’s expertise, animal diet, animal vendor, etc.) [17, 21].

The result of this painstaking effort was that three interventions that had been widely reported to be cardioprotective in numerous single-center studies (sildenafil, sodium nitrite, and chloramphenicol succinate) were found to be ineffective [18, 20] (Table). (Sodium nitrite had even been tested already in two very expensive clinical trials [16, 28]). Several other therapies, also reported to be cardioprotective in single-center studies, failed to reduce infarct size (Table). In contrast, ischemic preconditioning did reduce infarct size (thus serving as a positive control) [17]. Unfortunately, CAESAR’s life was too short to enable it to test a large number of interventions. NHLBI funding for the network covered less than 4 years of activity, and most of the life of the grant was spent in the formidable task of setting up this new infrastructure. Sadly, to date, CAESAR remains the only public network that has performed rigorous, multicenter testing of cardioprotective therapies proposed by external investigators. Albeit brief, its work has shown that the application of clinical trial-like rigor has a major impact on the results of preclinical studies (Table).

The most important mission of CAESAR, however, was not to test specific therapies, but to promote a fundamental change in the methodological approach of the cardiovascular community to cardioprotection, whereby the above-mentioned criteria of rigor would be widely incorporated in preclinical studies. Despite its relatively short life, it is gratifying to see that the seeds planted by CAESAR have germinated and produced fruit. Since the 2003 Bethesda meeting, things have really changed. A growing number of groups and investigators have advocated rigor in preclinical studies of cardioprotection [11–13, 19, 22, 25, 27, 29] as well as the establishment of a public registry of these studies [6]. Then, following the lead of *Circulation Research* in 2017 [2, 3], an increasing number of journals have started demanding adherence to rigor in preclinical studies. Perhaps the most comprehensive and authoritative set of rigor guidelines was published by Botker et al*.* in *Basic Research in Cardiology* in 2018 [5]. Modeled in part on CAESAR, the European Union has set up the CARDIOPROTECTION COST Action consortium (which, however, is funded only for communication and networking, not for projects) [10]. This “rigor revolution” is precisely what CAESAR hoped to trigger.

The paper by te Lintel Hekkert and colleagues in this issue of the Journal [30] is one of the best examples of the application of rigor to preclinical studies of cardioprotection. The authors sought to determine whether DMX-10001, a pro-drug of the MAP4K4 inhibitor MDX-5804, exerts cardioprotective actions in swine subjected to a 60-min coronary artery occlusion and 24 h of reperfusion. They found that infusion of DMX-10001, started 20 min before reperfusion and continued for 24 h, failed to reduce infarct size despite the fact that the plasma concentrations of the drug exceeded those previously found to limit the size of MI in mice and protect isolated human iPSC-derived cardiomyocytes in vitro [8].

The authors of this work should be congratulated for such an unusually thorough and rigorous preclinical test of cardioprotection. This study is a paradigm of what a preclinical study of infarct size reduction ought to be. It fully adheres to the criteria set forth in CAESAR [17, 21] and to the most recent guidelines [5], including submission to a preclinical registry (https://preclinicaltrials.eu) [6]. If studies of infarct size limitation had been conducted with this level of rigor over the last 50 years, there is little doubt that there would have been a dramatic reduction in “positive” results, a vast decrease in confusion and apparent disagreements at the preclinical level, as well as a marked decrease in premature and/or unwarranted clinical trials and attending expenses and false expectations. It is hoped that the publication of this study will further promote widespread adoption of the CAESAR [17, 21] and subsequent [5] criteria for rigor in preclinical investigations of putative cardioprotective agents.

The authors must also be lauded for not “pushing” their results. When infarct size was expressed as a percentage of left ventricular weight, there was a statistically significant 27% difference in favor of the treated group [30]. However, when infarct size was normalized to the size of the region at risk, the difference was no longer statistically significant. The aim of the study, declared prospectively, was to determine whether DMX-10001 reduces infarct size (normalized to risk region) by 20% of control or more, and so sample sizes were calculated to achieve a power of at least 80% to detect a 20% relative reduction. The observed relative reduction in normalized infarct size averaged 13% (*P* = 0.08), and although it is possible that with more animals this difference may have become statistically significant, the endpoint of the study was not achieved and the authors appropriately conclude that the drug failed to reduce infarct size. This level of objectivity is what the field of cardioprotection needs.

The protocol used in this study was immaculate. It sets a standard for future studies of similar nature. The main lingering question is whether DMX-5804 inhibits MAP4K4 in pigs. Although the drug has been shown to be effective in other species, there are no data in porcine tissues or cells. A second, minor comment relates to the studies of left ventricular performance at 24 h after reperfusion. Given the extensive presence of myocardial stunning at this time, it is very unlikely that an improvement in left ventricular function, particularly in global function, would be observed even if there was a significant reduction in infarct size. That the outcome of this experiment did not confirm earlier studies in mice and isolated myocytes [8] is not surprising; given the enormous and obvious differences between these models and larger mammals, it seems logical to expect that results obtained in the former may not be applicable to the latter. Although large animal studies are appreciated less and less by reviewers and granting agencies, there is no circumventing them; it would be irrational to attempt clinical trials without supporting evidence in large mammals.

The study by Te Lintel Hekkert et al. [30] is one of the best preclinical studies of infarct size reduction that has appeared in the literature thus far. It is the kind of study that CAESAR aimed at promoting. It is hoped that future investigations will continue to adhere to this level of rigor, for this is the only way forward. Indeed, it is only with this level of rigor that the confusion that dominates the field can be cleared and the truth regarding the efficacy of cardioprotective therapies can be attained.

**Therapies tested in the CAESAR consortium**

**Table:** 

Therapy	Species	Effect on Infarct Size
Ischemic preconditioning	Mouse Rabbit Pig	↓ ↓ ↓
Sildenafil	Mouse Rabbit Pig	No effect No effect No effect
Sodium nitrite	Mouse Rabbit Pig	No effect No effect No effect
Chloramphenicol succinate	Pig	No effect
Mab	Rabbit	No effect
Putative cardioprotective drug 1	Mouse	No effect
Putative cardioprotective drug 2	Mouse	No effect
Putative cardioprotective drug 3	Mouse	No effect
